# PCR performance of a thermostable heterodimeric archaeal DNA polymerase

**DOI:** 10.3389/fmicb.2014.00195

**Published:** 2014-05-07

**Authors:** Tom Killelea, Céline Ralec, Audrey Bossé, Ghislaine Henneke

**Affiliations:** ^1^Université de Bretagne Occidentale, UMR 6197, Laboratoire de Microbiologie des Environnements ExtrêmesPlouzané, France; ^2^Ifremer, UMR 6197, Laboratoire de Microbiologie des Environnements ExtrêmesPlouzané, France; ^3^CNRS, UMR 6197, Laboratoire de Microbiologie des Environnements ExtrêmesPlouzané, France

**Keywords:** DNA polymerase, Archaea, family D, PCR, *Pyrococcus*

## Abstract

DNA polymerases are versatile tools used in numerous important molecular biological core technologies like the ubiquitous polymerase chain reaction (PCR), cDNA cloning, genome sequencing, and nucleic acid based diagnostics. Taking into account the multiple DNA amplification techniques in use, different DNA polymerases must be optimized for each type of application. One of the current tendencies is to reengineer or to discover new DNA polymerases with increased performance and broadened substrate spectra. At present, there is a great demand for such enzymes in applications, e.g., forensics or paleogenomics. Current major limitations hinge on the inability of conventional PCR enzymes, such as *Taq*, to amplify degraded or low amounts of template DNA. Besides, a wide range of PCR inhibitors can also impede reactions of nucleic acid amplification. Here we looked at the PCR performances of the proof-reading D-type DNA polymerase from *P. abyssi*, Pab-polD. Fragments, 3 kilobases in length, were specifically PCR-amplified in its optimized reaction buffer. Pab-polD showed not only a greater resistance to high denaturation temperatures than *Taq* during cycling, but also a superior tolerance to the presence of potential inhibitors. Proficient proof-reading Pab-polD enzyme could also extend a primer containing up to two mismatches at the 3' primer termini. Overall, we found valuable biochemical properties in Pab-polD compared to the conventional *Taq*, which makes the enzyme ideally suited for cutting-edge PCR-applications.

## Introduction

On the basis of their amino acid sequence and structural analysis, DNA polymerases have been classified into seven families, A, B, C, D, E, X, and Y (Delarue et al., 1990; Braithwaite and Ito, 1993; Joyce and Steitz, 1994; Cann et al., 1998; Ishino et al., 1998; Ohmori et al., 2001; Lipps et al., 2003). Despite the conserved template-directed synthesis (or editing) of a complementary deoxyribonucleotide chain (Kornberg and Baker, 1992; Hubscher et al., 2010) and the similarity of the three-dimensional organization of their polymerase domain (“palm,” “thumb,” and “finger”) (Joyce and Steitz, 1994; Rothwell and Waksman, 2005), DNA polymerases differ extensively in many of their specific features (e.g., processivity, fidelity, rate of DNA synthesis, and nucleotide selectivity) (Hubscher et al., 2010; Langhorst et al., 2012).

Beginning with the discovery and characterization of DNA polymerase I (Family A) from *Thermus aquaticus* (*Taq*) (Chien et al., 1976), a variety of thermostable DNA polymerases have been isolated and identified from prokaryotic organisms. Besides their crucial biological functions, thermostable DNA polymerases have proven to be technically and economically important enzymes. They are versatile tools used in DNA technologies such as cycle sequencing and polymerase chain reaction (PCR) (Pavlov et al., 2004). Since its invention by Saiki et al. (1985), PCR has become a widespread molecular biology method. Originally PCR was developed to specifically amplify a stretch of DNA prior to cloning; however, its flexibility underpins a number of applications such as site-directed mutagenesis, genetic diagnostics, gene therapy, forensics, and paleogenomics.

In the PCR, DNA amplification is performed by thermostable enzymes; invariably either family A DNA polymerases from thermophilic and hyperthermophilic Bacteria (e.g., *Thermus aquaticus*, Taq-polA and *Thermotoga maritima*, Tma-polA) or family B DNA polymerases from hyperthermophilic Archaea (e.g., *Pyrococcus furiosus*, Pfu-polB and *Pyrococcus abyssi*, Pab-polB; *Isis*™). Family Y DNA polymerase from the hyperthermophilic archaeon *Sulfolobus solfataricus*, Sso-polY, is also an enzyme marketed for PCR, but with specialist applications (McDonald et al., 2006). Each thermostable DNA polymerases has different characteristics (e.g., thermostability, processivity, fidelity, specificity, modified nucleotides selection, resistance to contaminants and inhibitors, slippage, pyrophosphorolysis) and to achieve optimal results, the choice of a PCR enzyme depends on the application itself (e.g., high-yield PCR, high-fidelity PCR, routine PCR, multiplex PCR, colony PCR, difficult PCR, long Range PCR, fast PCR, incorporation of modified nucleotides). Detailed information about individual properties of PCR enzymes and their related applications have been recently reviewed (Terpe, 2013).

Archaeal family B DNA polymerases are generally more thermostable enzymes than bacterial polymerases (Kong et al., 1993; Takagi et al., 1997; Cambon-Bonavita et al., 2000; Gueguen et al., 2001; Hogrefe et al., 2001; Moussard et al., 2006; Marsic et al., 2008). In addition, when accuracy is a desired property it leads to a preference for archaeal enzymes which possess a 3'–5' proof-reading exonuclease activity, absent in most thermostable bacterial polymerases (Eckert and Kunkel, 1991; Cline et al., 1996; Perler et al., 1996). On the other hand, archaeal family B DNA polymerases can incorporate dUTP during DNA replication but cannot copy these strands in subsequent DNA amplification rounds (Fogg et al., 2002). With constant development of new techniques based on PCR, improved DNA polymerase variants are continuously being engineered including: polymerases which are thermo-activated (hot start polymerases) (Sharkey et al., 1994), bacterial and archaeal DNA polymerase derivatives with increased processivity (Wang et al., 2004), archaeal family B DNA polymerase variants insensitive to uracil inhibition (Fogg et al., 2002), or thermostable enzymes proficient in the synthesis of fluorescent CyDNAs (Ghadessy et al., 2004; Wynne et al., 2013). Error-prone PCR which creates random mutagenesis of the parental gene relies on different strategies using either low-fidelity DNA polymerase variants (Biles and Connolly, 2004) or error-prone PCR conditions (McCullum et al., 2010; Le et al., 2013). Most of these conventional marketed PCR enzymes have the drawback of replicating exclusively native DNAs. Among thermostable DNA polymerases that can counteract this major limitation, archaeal translesional family Y DNA polymerases are of particular interest. They are able to bypass a variety of DNA lesions and therefore are well-suited for the PCR amplification of ancient and damaged DNAs (McDonald et al., 2006).

Over 14 years ago a new family of archaeal DNA polymerases (the D-family) was discovered (Uemori et al., 1997). Despite obvious interest in the biochemical characteristics of archaeal family D DNA polymerases (Cann et al., 1998; Gueguen et al., 2001) there is limited information regarding the structure (Yamasaki et al., 2010; Matsui et al., 2011) and kinetics of these enzymes (Palud et al., 2008; Richardson et al., 2013a). However, it is known that these thermostable DNA polymerases are heterodimeric and comprise a small subunit (DP1), possessing 3' → 5' exonuclease activity, and a large subunit (DP2), exhibiting DNA polymerase activity (Cann and Ishino, 1999). The small subunit shares low level of homology with the non-catalytic B-subunits of the eukaryotic family B DNA polymerases (Cann et al., 1998; Ishino et al., 1998; Gueguen et al., 2001). In contrast, the sequence of the large subunit shows no significant homology to any other DNA polymerase (Macneill et al., 2001). Currently, the growing body of evidence suggests involvement of the family D DNA polymerases in genome replication in Archaea (Henneke et al., 2005; Rouillon et al., 2007; Castrec et al., 2009; Cubonova et al., 2013).

Family D DNA polymerases from hyperthermophilic Archaea which have been biochemically characterized, to date, are from the Pyrococcus genus such as *Pyrococcus horikoshii* (Shen et al., 2001), *Pyrococcus furiosus* (Uemori et al., 1997), and *Pyrococcus abyssi* (Gueguen et al., 2001). These microorganisms contain only one family B enzyme in addition to the family D DNA polymerase. In contrast with commercialized family B enzymes (Pfu-polB and Pab-polB), none of the family D DNA polymerases have been reported as active enzymes in PCR or in other DNA technologies.

Family D DNA polymerase from *Pyrococcus abyssi* shows comparable nucleotide selectivity to family B, and increased fidelity with the active proofreading (Palud et al., 2008; Richardson et al., 2013a). Family D DNA polymerase preferentially binds to primer/template with an affinity higher than family B, while showing reduced DNA synthesis of smaller DNA fragments (Henneke et al., 2005). The assembly of the two subunits into a heterodimer is required to substantially increase both polymerase and exonuclease activities in family D, while both activities are contained within the same polypeptide in the family B DNA polymerase (Castrec et al., 2010; Gouge et al., 2012). These functional properties suggest that family D DNA polymerase might perform PCR performance distinct than Pab-polB. In this paper, the ability of the recombinant family D DNA polymerase from *Pyrococcus abyssi* (Pab-polD) to PCR-amplify DNA has been developed in terms of biochemical and PCR performance parameters (e.g., stability to heat denaturation steps, extension efficiency, resistance to common PCR inhibitors). These results are compared with data acquired from commercial thermostable DNA polymerases (Pab-polB and Taq-polA) and reveal that family D DNA polymerase has significant commercial value in PCR technology.

## Materials and methods

### Chemicals and enzymes

Unlabeled dNTPs were purchased from MP Biomedicals. Pab-polD was cloned, expressed, and purified as described (Henneke et al., 2005). One unit of Pab-polD corresponds to the incorporation of 1 nmol of total dTMP into acid precipitable material per minute at 65°C in a standard assay containing 0.5 mg (nucleotides) of poly(dA)/oligo(dT)_10:1_. Pab-polB (*Isis* DNA polymerase) and Taq-polA (*Taq* DNA polymerase) were purchased from MP biomedicals. All other chemicals and bioreagents were analytical grade and purchased from Sigma-Aldrich (St. Louis, MO). Bioactive small molecules (Human hemoglobin, humic acid, hematin, heparin, and urea) were molecular biology grade from Sigma-Aldrich (St. Louis, MO). The 1.7 million base-pair genome of *Pyrococcus abyssi* GE5 was obtained as described (Charbonnier et al., 1995).

### Polymerase chain reaction (PCR enzymes)

PCR primers for the amplification of targets in genomic DNA from *P. abyssi* (*Pab*) genomic sequence from 1323272 to 1333272 base pairs (bp) were purchased from Eurogentec (Belgium). The primer sequences, the *Pab* genomic sequence, and the size of the expected amplicons (in kilobases, kb) are summarized in Table 1. These selective amplifications were dictated by the availability of total genomic DNA from *P. abyssi* devoid of any potential PCR inhibitors and the use of thermally stable oligonucleotide primers. PCR performance parameters of Pab-polD were determined in the optimized buffer: 20 mM Tris-HCl pH 9, 25 mM KCl, 10 mM (NH_4_)_2_SO_4_, 2 mM MgCl_2_, 0.1 mg/ml Bovine Serum Albumin (BSA), 0.1% (v/v) Tween 20. PCR reactions (25 μl) contained 200 nM of each primer, 200 μM dNTPs, and 100 ng of genomic DNA unless otherwise specified. The PCR conditions for commercial Taq-polA and Pab-polB were set according to the manufacturers' instructions. All reactions were run in (at least) duplicate. Negative control included all reaction components without genomic DNA. The amplification was carried out in GeneAmp® PCR System 9700 Thermal Cycler (Applied Biosystems) and in Veriti® 96-Well Thermal Cycler (Applied Biosystems). Cycling conditions were 2 min at 94°C; 30 cycles with 1 min denaturation at 94°C, 1 min annealing at 58°C and extension at 72°C at the indicated times. A final extension step at 72°C was applied before the termination of the reaction as specified in the corresponding figure legends. Elongation temperature was set at 72°C according to the manufacturer protocols for Taq-PolA and Pab-polB, therefore validating the temperature of assay performance by Pab-polD. The products were analyzed with 1% agarose gel electrophoresis, stained with ethidium bromide, and visualized with the Molecular Imager FX (BioRad). When mentioned, activity (%) is expressed as a percentage of the maximal value obtained in each experiment.

**Table 1:** **Primers applied in this study**.

**Primer name**	**Target length (kb)**	**Primer sequence (5'–3')**	***Pab* genomic sequence (bp)**	**GC % of DNA target**
Start_F		GCAAAGGAGTTTGCCCAGCTTATAGAGACGGCC	1333240–1333272	
Start_1M_F		GCAAAGGAGTTTGCCCAGCTTATAGAGACGGCA	1333240–1333272	
Start_2M_F		GCAAAGGAGTTTGCCCAGCTTATAGAGACGGAA	1333240–1333272	
Start_3M_F		GCAAAGGAGTTTGCCCAGCTTATAGAGACGCAA	1333240–1333272	
Start_4M_F		GCAAAGGAGTTTGCCCAGCTTATAGAGACCCAA	1333240–1333272	
0.5_R	0.5	TCAACCTCCTGGGTTTCACCTTCGGCCCTC	1332773–1332802	47.2
1_R	1.1	CCTCAGCCTCAACGCTTATTGGTTCCCCGT	1332173–1332202	47.1
2_R	1.95	GAAGATTAAGGAGAGGGGGAGGGCAAAGGCTGGTGG	1331322–1331357	46.8
3_R	2.95	CGAGGAAGCTAGAGGAGAGGGGTGCTGAGTGC	1330322–1330353	47.5
4_R	4.15	GGAGGGGCAACTACTATGGATACCACCCTTCC	1329122–1329153	46.7
10_R	10	TCCAAGAGTTCCTCCCTGGGGAACGGCCTGAACC	1323273–1323306	47

F and R denote forward and reverse primers, respectively. M stands for mismatch.

PCR experiments in the presence of inhibitors were conducted with the optimized Pab-polD buffer as described above. The PCR conditions for Pab-polB and Taq-polA were set according to the manufacturers' instructions. A 0.5 kb fragment was amplified from *Pab* genomic DNA using the 500 bp reverse and forward primers (listed in Table 1). Titration of each inhibitor was performed at least in triplicate. Cycling conditions were 2 min at 94°C; 30 cycles with 1 min denaturation at 94°C, 1 min annealing at 58°C, and 2 min extension at 72°C; final extension, 5 min at 72°C. The products were analyzed with 1% agarose gel electrophoresis, stained with ethidium bromide, and visualized with the Molecular Imager FX (BioRad).

## Results

### Optimized PCR reaction conditions

**Figure 1:**
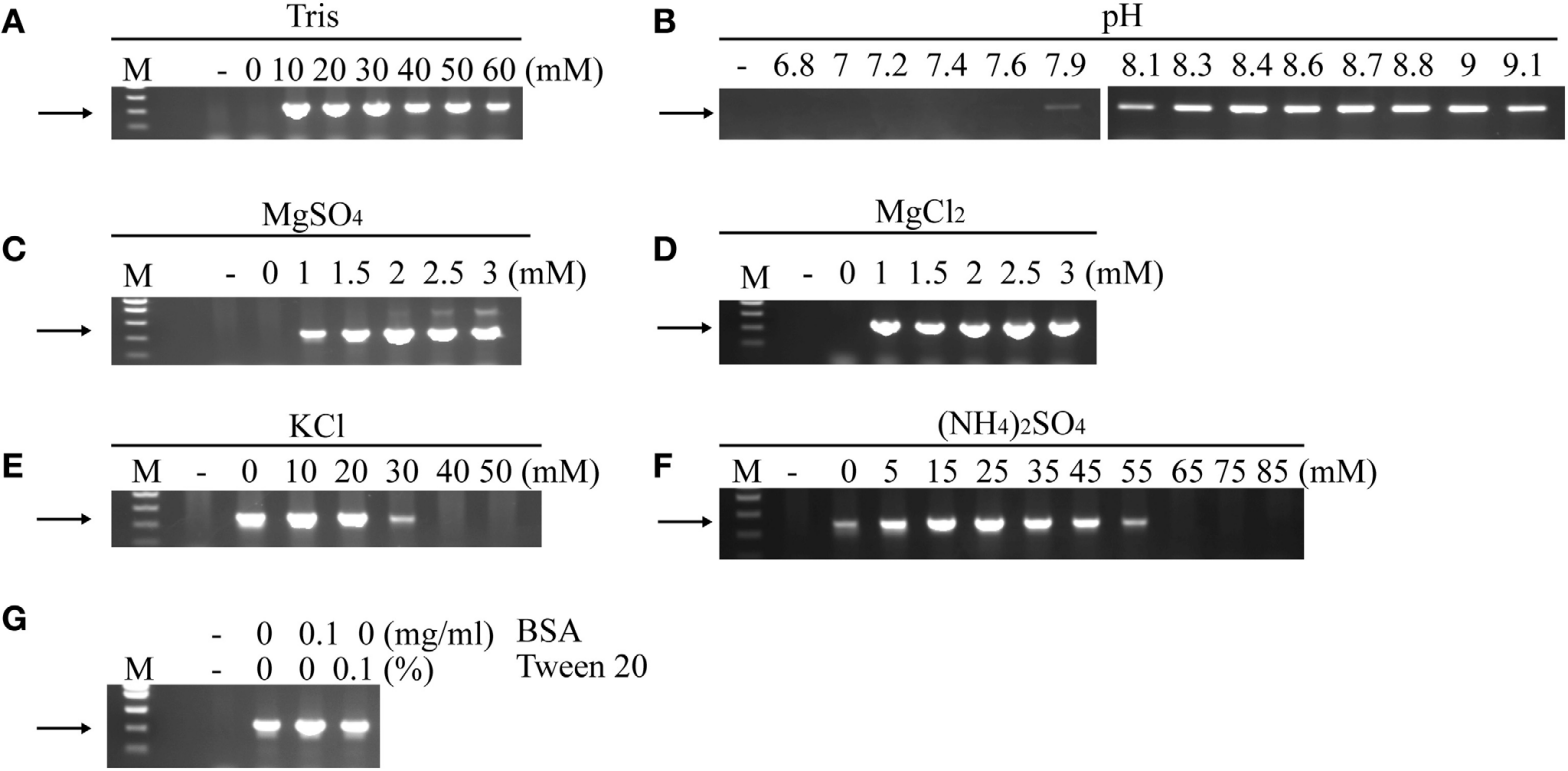
**Effect of buffer concentration, pH, salts, ions, BSA, and Tween 20 on PCR amplification of the 0.5 kb target by Pab-polD**. PCR reactions were carried out using 100 ng of genomic DNA with 0.1 U of Pab-polD. The optimized reaction buffer was used as is shown in Table 2, except for the variable condition, which was altered as shown in the figure. PCR program was (2 min at 94°C) × 1; (1 min at 94°C, 1 min at 58°C, 2 min at 72°C) × 30; (5 min at 72°C) × 1. Effect of varying the Tris concentration **(A)**, pH **(B)**, MgSO_4_ **(C)**, MgCl_2_ **(D)**, KCl **(E)**, (NH_4_)_2_SO_4_ **(F)**. The absence or presence of BSA and Tween 20 are shown in **(G)**. Molecular weight markers (M) are SmartLadder SF from Eurogentec. (–) denotes negative control without Pab-polD. The arrow indicates the specific 0.5 kb band.

The optimized buffer for PCR with Pab-polD was obtained by varying different components of the standard Pab-polB reaction buffer. Pab-polD PCR activity was optimal in 10–30 mM Tris-HCl buffer concentration (Figure 1A) and between pH 8.3 and 9 (measured at 25°C) (Figure 1B). Incubation of Pab-polD with either magnesium chloride (MgCl_2_) or magnesium sulfate (MgSO_4_) in the same concentration range resulted in the amplification of non-specific and undesirable PCR products with MgSO_4_ (Figure 1C). Reactions carried out with MgCl_2_ gave rise to the amplification of specific products in the optimal concentration range tested (Figure 1D). The effects of different salt concentrations of potassium chloride (KCl) (Figure 1E) and ammonium sulfate ((NH_4_)_2_SO_4_) (Figure 1F) were analyzed in PCR by Pab-polD. The maximal activity detected with the two salts was 0–20 mM for KCl and 15–25 mM for (NH_4_)_2_SO_4_, respectively. Although the presence of (NH_4_)_2_SO_4_ further enhanced PCR amplification at optimal concentration, KCl could be dispensable. Finally, the two additives, Tween and BSA, used in the standard Pab-polB reaction buffer were added or omitted in Pab-polD PCR reactions. In the conditions tested, 0.1 mg/ml BSA and 0.1% Tween did not significantly improved the amount of PCR products by Pab-polD (Figure 1G). Overall, the optimal reaction buffer for *in vitro* amplification of DNA fragments by Pab-polD has been determined and is now available in Table 2.

**Table 2:** **Properties of experimental thermophilic DNA polymerases**.

	**Pab-polD**	**Pab-polB**	**Taq-polA**
Name	Pab-polD	*Isis*	*Taq*
Microorganism	*Pyrococcus abyssi*	*Pyrococcus abyssi*	*Thermus aquaticus*
Pol family	D	B	A
Half-life	90°C/50 min (Gueguen et al., 2001)	100°C/5 h	97.5°C/9 min (Lawyer et al., 1993; Perler et al., 1996)
	80°C/2 h (Gueguen et al., 2001)	90°C/10 h (Gueguen et al., 2001; Dietrich et al., 2002)	
Error rate × 10^−6^ (/bp/duplication)	N.P.	0.66 (Dietrich et al., 2002)	24 (Dietrich et al., 2002)
Reaction buffer	20 mM Tris-HCl, pH (25°C) 9.0, 25 mM KCl, 10 mM (NH4)_2_SO_4_, 2 mM MgCl_2_, 0.1% Tween 20, 0.1 mg/ml BSA	20 mM Tris-HCl, pH (25°C) 9.0, 25 mM KCl, 10 mM (NH4)_2_SO_4_, 1.5 mM MgSO_4_, 0.1% Tween 20, 0.1 mg/ml BSA	10 mM Tris-HCl pH (25°C) 9.0, 50 mM KCl, 1.5 mM, MgCl_2_, 0.2 mg/mL BSA
Exonuclease activities	3'–5' (Gueguen et al., 2001)	3'–5' (Gueguen et al., 2001)	5'–3' (Lawyer et al., 1993)
Extension rate: kb/min (nt/s)	0.33 (5.5)	0.48 (8)	0.39 (6.5)

N.P., Not Published.

Error rate for Pab-polB and Taq-polA were determined in the same experiments under the same conditions (Dietrich et al., 2002).

### Effect of input genomic DNA on PCR efficiency and specificity

**Figure 2:**
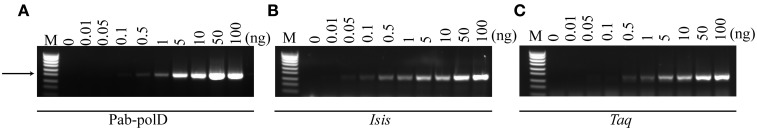
**Effect of input genomic DNA on PCR efficiency and specificity**. PCR amplification of the 0.5 kb target (Table 1) was carried out with 0.1 U of Pab-polD **(A)**, 1 U of Pab-polB **(B)**, and 1 U of Taq-polA **(C)** in their respective reaction buffer (Table 2). PCR program was (2 min at 94°C) × 1; (1 min at 94°C, 1 min at 58°C, 2 min at 72°C) × 30; (5 min at 72°C) × 1. Molecular weight markers (M) are SmartLadder SF from Eurogentec. The arrow indicates the specific 0.5 kb band.

PCR amplification, targeting the 0.5 kb fragment in the 1.7 million base-pair genome of *P. abyssi* (Table 1), was employed to determine the minimal amount of DNA required. In its optimal reaction conditions, Pab-polD was able to specifically amplify the 0.5 kb target from 0.5 to 100 ng of input genomic DNA (Figure 2A). Although the yield of PCR products was severely reduced at 0.5–1 ng, all three enzymes retained polymerase activity (~2–5% of activity) (Figures 2A–C). In the presence of 0.1 ng, only Pab-polB was capable of amplification of the 0.5 kb DNA target (Figure 2B).

### Impact of thermal denaturation during cycling

**Figure 3:**
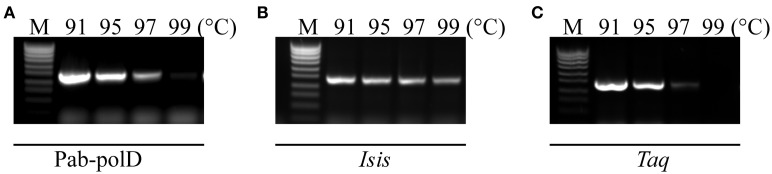
**Impact of thermal denaturation during cycling**. PCR amplification of the 0.5 kb target (Table 1) was carried out with 0.1 U of Pab-polD **(A)**, 1 U of Pab-polB **(B)**, and 1 U of Taq-polA **(C)** in their respective reaction buffer (Table 2). PCR program was (2 min at 94°C) × 1; (1 min at the indicated temperature, 1 min at 58°C, 2 min at 72°C) × 30; (5 min at 72°C) × 1. Molecular weight markers (M) are SmartLadder SF from Eurogentec.

The resistance of Pab-polD to the temperature of the denaturation step during cycling was investigated in comparison with Taq-polA and Pab-polB. PCR amplifications of the 0.5 kb DNA target were performed with 4 different thermal denaturation steps during cycling (91, 95, 97, and 99°C). As shown in Figure 3A, Pab-polD could yield specific PCR products (15% activity compared to 91°C) when the denaturation temperature was as high as 97°C. PCR products were hardly detectable at 99°C. Interestingly, the PCR efficiency of Pab-polB was not profoundly affected by the increase in the thermal denaturation step during cycling, while Taq-polA was mostly inactive above 95°C (Figures 3B,C). Taken together, these results are in agreement with those published previously (Dietrich et al., 2002) and indicate that hyperthermophilic *Pab* DNA polymerases are more robust than the thermophilic Taq-polA.

### Rate of DNA extension

**Figure 4:**
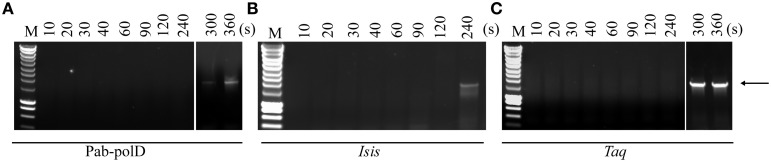
**Rate of DNA extension**. PCR amplification of the 1.95 kb target (Table 1) was carried out with 0.1 U of Pab-polD **(A)**, 1 U of Pab-polB **(B)**, and 1 U of Taq-polA **(C)** in their respective reaction buffer (Table 2). PCR program was (2 min at 94°C) × 1; (1 min at 94°C, 1 min at 58°C, varying times in seconds as indicated at 72°C) × 30. Molecular weight markers (M) are SmartLadder LF from Eurogentec. The arrow indicates the specific 1.95 kb band.

In order to determine the rate of primer extension by Pab-polD, the 1.95 kb DNA target was amplified using variants of the end-point PCR method employed throughout this study. Numerous reactions were carried out, with each PCR possessing an incrementally larger extension time than the last, until end-point PCR products were detectable on an agarose gel. Our initial attempt, in which the longest extension time was set to 240 s, failed with Pab-polD and Taq-polA (Figures 4A,C). However, the 1.95 kb DNA target was successfully amplified by Pab-polB (Figure 4B). Pab-polD exhibited a lower extension rate than Taq-polA since a single specific product at the expected size appeared at 360 and 300 s, respectively for each enzyme. According to these results, the rate of extension by Pab-polD was estimated at 0.33 kb/min, resembling that of Taq-polA (0.39 kb/min), but dissimilar to that of Pab-polB (0.48 kb/min) (Table 2).

### PCR amplification of DNA fragments of various lengths

**Figure 5:**
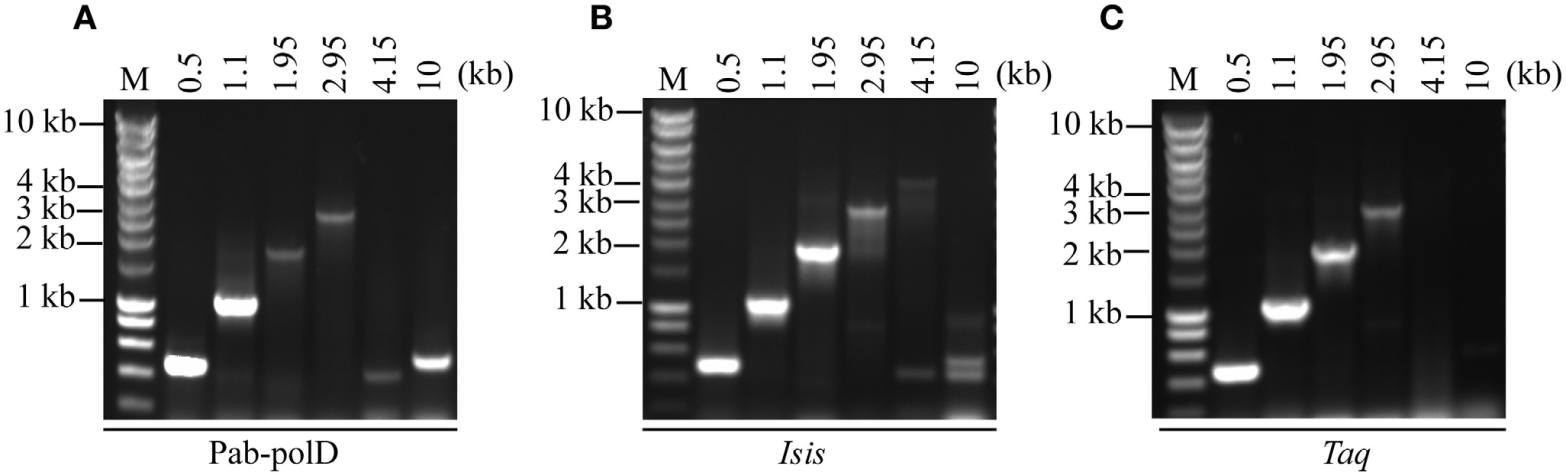
**PCR amplification of DNA fragments of various lengths**. PCR reactions were carried out using 100 ng of genomic DNA with 0.1 Uof Pab-polD **(A)**, 1 U of Pab-polB **(B)**, and 1 U of Taq-polA **(C)** in their respective reaction buffer (Table 2). Primer sets were chosen to amplify 0.5, 1.1, 1.95, 2.95, 4.15, and 10 kb (Table 1). PCR programs were (2 min at 94°C) × 1; (1 min at 94°C/1 min at 58°C/2, 4, 6, 8, 11, and 16 min with respect to the target length at 72°C) × 30. Molecular weight markers (M) are SmartLadder LF from Eurogentec.

To determine the ability of Pab-polD to amplify various sized target sequences (ranging 0.5 to 10 kb) from genomic DNA, six specific primers have been designed (Table 1). The extension time assigned to the amplification of each DNA fragment during cycling is in agreement with the extension rate of Pab-polD described above. In the range of 0.5–1.1 kb, Pab-polD and Taq-polA efficiently and specifically amplified DNA fragments (Figures 5A,C). Amplification of DNA molecules ranging from 1.95 to 2.95 kb was severely reduced for Pab-polD and to lower extent for Taq-polA (~8% activity for the 2.95 kb target compared with the 0.5 kb target for both DNA polymerases), with bands at these sizes being faint and slightly detectable in ethidium bromide stained agarose gels after 30 PCR cycles. Amplification of target sequences ranging from 4.15 to 10 kb by Pab-polD and Taq-polA failed in the conditions tested (Figures 5A,C), with Pab-polD producing weak and unspecific bands. With the exception of the 4.15 and 10 kb DNA targets, Pab-polB amplified single products with the expected size (Figure 5B). Except for the 10 kb DNA target, a single product was amplified with Pab-polB. However, the yield of specific PCR products is decreased for the 2.95 kb DNA target. The above data clearly show that Pab-polD is suitable for the specific amplification of DNA molecules in the range of 0.5–2.95 kb, while showing reduced yields above 1.1 kb. Although not shown, replacement of Pab-polD optimal buffer conditions by Taq-polA or Pab-polB reaction buffer did not improve the yield of amplified 1.95–2.95 kb DNA targets, nor enhanced the synthesis of longer PCR products.

### PCR amplification in the presence of primer mismatches

**Figure 6:**
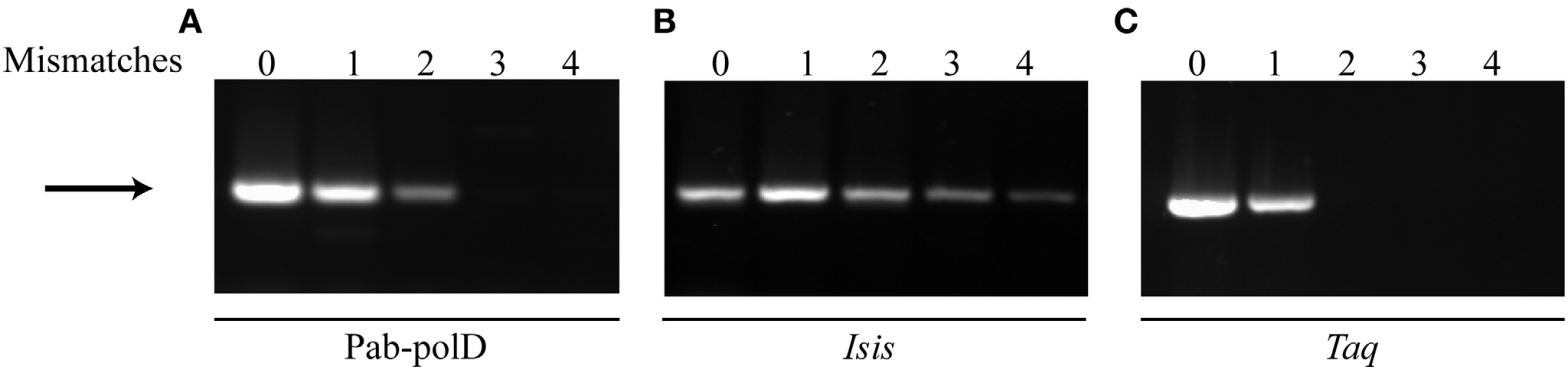
**PCR amplification in the presence of primer mismatches**. PCR amplification of the 0.5 kb target (Table 1) was carried out with 0.1 U of Pab-polD **(A)**, 1 U of Pab-polB **(B)**, and 1 U of Taq-polA **(C)** in their respective reaction buffer (Table 2). Primer sets were chosen to introduce 0, 1, 2, 3, and 4 mismatches at the 3'-termini of the forward primer (Table 1). PCR program was (2 min at 94°C) × 1; (1 min at 94°C, 1 min at 58°C, 2 min at 72°C) × 30; (5 min at 72°C) × 1. The arrow indicates the specific 0.5 kb band.

Complete 3'-terminal primer annealing to its complementary target sequence is a very important factor for the success and stringency of PCR (Petruska et al., 1988; Ishii and Fukui, 2001; Sipos et al., 2007). To evaluate the impact of primer mismatches on PCR efficiency and specificity by Pab-polD, forward primer sets containing up to four mismatches at the 3'-end have been designed for full amplification of the 0.5 kb DNA target from genomic DNA (Table 1). As shown in Figure 6A, specific amplification of the 0.5 kb DNA target could be achieved in the presence of either one or two 3'-end terminal mismatches, with lower PCR efficiency observed with two mismatches (~5% remaining activity). The presence of three or four mismatches had a detrimental effect on the extension efficiency by Pab-polD. Taq-polA DNA polymerase generated specific PCR products with only one mismatched primer termini and longer mismatches prevented successful PCR amplification (Figure 6C). Although strand extension, and hence PCR amplification efficiency, were influenced by multiple mismatches at the 3' end of the primer, the detection of specific PCR products was never compromised for Pab-polB (Figure 6B). Here, the data pointed out that Pab-polD is a suitable enzyme for Taq-polA substitution when 3'-terminal mismatched primers are refractory to PCR amplification.

### PCR amplification in the presence of known PCR inhibitors

To investigate the impact of known PCR inhibitors (Al-Soud and Radstrom, 1998, 2001; Schrader et al., 2012), the 0.5 kb target was amplified from genomic DNA in reactions containing various levels of PCR-inhibiting compounds. The results are presented in Table 3. It was found that the variation of the ionic strength affected the PCR performances of the three different DNA polymerases. Indeed, Pab-polD was more resistant to NaCl ions than were Pab-polB and Taq-polA. In the conditions tested, a specific PCR product was detectable at the permissive concentration of 50 mM with Pab-polD, while absent with Pab-polB and Taq-polA (Supplementary Figure 1). When SDS (Sodium Dodecyl Sulfate), the anionic reagent well-known for its protein-denaturing effects, was employed at a concentration of 0.02%, PCR amplification was successful with Pab-polD and Pab-polB (Table 3); however, the yield of PCR products was dramatically impaired (Supplementary Figure 1). In comparison, Taq-polA was weakly active at a SDS concentration of 0.01%. CaCl_2_, a potent PCR inhibitor (Bickley et al., 1996; Al-Soud and Radstrom, 1998) found for instance in milk, cheese, or bones, was found to impede the amplification of the specific 0.5 kb target when incubated at a concentration of 2 mM for Pab-polD and Taq-polA (Table 3). Family B DNA polymerase was highly sensitive to CaCl_2_ as observed the severe reduction in PCR activity at a final concentration of 1 mM (Supplementary Figure 1).

**Table 3:** **Potential inhibitory effects of organic and inorganic substances on PCR**.

	**Inhibitory concentration of compounds**		
	**Pab-polD**	**Pab-polB**	**Taq-polA**
NaCl (mM)	>50	>25	>25
SDS % (w/v)	>0.02	>0.02	>0.01
CaCl_2_ (mM)	>2	>1	>2
Hemoglobin (mg/ml)	>13	>13	>3.5
Hematin (μM)	>12.5	>25	>12.5
Heparin (U/ml)	–	–	–
EDTA (mM)	>0.5	>0.5	>0.5
Urea (mM)	>25	>100	>50
Humic acid (ng/μl)	–	>62	>15
Phenol % (v/v)	>0.6	>0.8	>0.4
Ethanol % (v/v)	>4	>5	>4
Isopropanol % (v/v)	>2	>8	>2

It has been reported that heme compounds, for instance hemoglobin and hematin, yielded interferences in PCR amplifications (Akane et al., 1994). For these reasons, we investigated Pab-polD PCR performances in the presence of these two blood-substances. Interestingly, hemoglobin never altered PCR specificity and efficiency by Pab-PolD and Pab-polB even at a final concentration of 13 mg/ml (Supplementary Figure 1). However, PCR performances by Taq-polA were entirely compromised at the minimal concentrations of 1.75–3.5 mg/ml. Hematin decreased the yield of PCR products by Pab-polD and Taq-polA when present at a final concentration of 12.5 μM, while Pab-polB still retained significant activity in the presence of >25 μM of hematin.

The blood anticoagulant substance heparin described to inhibit PCR (Yokota et al., 1999) has been investigated. In the PCR reactions, the addition of heparin suppressed the formation of a specific PCR product in a dose-dependent fashion (from 0.006 to 0.2 U/μl) for all three PCR enzymes. Moreover, EDTA (EthyleneDiamineTetraAcetic acid), used also as a common anticoagulant to treat blood samples, and included in several elution buffers of nucleic acid purification, has been described to interfere with PCR (Yokota et al., 1999). In this study, EDTA had an inhibitory effect at a concentration greater than 0.5 mM for all three PCR enzymes. This result is likely indicative of the chelation of Mg^2+^ ions present in reaction buffers of each DNA polymerase, therefore compromising DNA amplification.

Since urea has been identified as the main component of urine that inhibits PCR (Khan et al., 1991), we challenged Pab-polD, Pab-polB, and Taq-polA in its presence. As shown in Table 3, Pab-polB was the most resistant DNA polymerase, specifically amplifying the 0.5 kb target even at the highest concentration of 100 mM. Pab-polD and Taq-polA showed inhibitory concentrations >25 and 50 mM, respectively. Humic acid, representative of environmental samples (e.g., soil, water, and dead matter), has been recognized as an efficient PCR inhibitor even at low concentrations (Tsai and Olson, 1992; Ijzerman et al., 1997). For this reason, all three enzymes have been submitted to PCR amplification with increasing amounts of humic acid (15–250 ng/μl). No PCR product was visible with Pab-polD, even at the lowest concentration. PCR amplification with Taq-polA was positive up to a humic acid concentration of 15 ng/μl. Pab-polB was the most resistant since it could PCR amplify the 0.5 kb target at 62.5 ng/μl of humic acid (Table 3).

Phenol, ethanol, and isopropanol are common organic substances used in the procedure for genomic extraction (Charbonnier et al., 1995) and relevant of food and environmental samples (Wilson, 1997). Interestingly, PCR performances of family B DNA polymerase were never affected by the varying concentrations of the three organic compounds (Supplementary Figure 1). In contrast, Taq-polA and Pab-polD exhibited similar inhibitory concentrations of isopropanol and ethanol (>2 and >4%, respectively). Moreover, Pab-polD was more tolerant to PCR inhibition by phenol than Taq-polA (Table 3). Overall, this comparative study clearly revealed that Pab-polB is the most tolerant enzyme to PCR inhibitors. Pab-polD seems to have a higher resistance to particular PCR inhibitors than Taq-polA (e.g., NaCl, SDS, Phenol, and hemoglobin), although sharing similar sensitivity (EDTA, ethanol, isopropanol, calcium, hematin, heparin). Finally, Taq-polA exhibited superior resistance to humic acid and urea than Pab-polD.

## Discussion

The family D DNA polymerase from *P. abyssi* has been applied to PCR on genomic DNA and submitted to varying chemical parameters in order to evaluate its performance. For this purpose, a buffer has been optimized using the Pab-polB reaction buffer as a starting point due to this family B DNA polymerase originated from the same archaeon, *P. abyssi*. Here, we show for the first time that a family D DNA polymerase is functional in PCR amplification of a 0.5 kb DNA target. Under the conditions listed in Table 1, the three enzymes were analyzed to compare thermal resistance. Pab-polD was identified as more resistant than Taq-polA, yet not as robust as Pab-polB to the increased thermal denaturation during cycling (Table 2). These data obtained for the commercial enzymes being comparable with that already published (Gueguen et al., 2001; Dietrich et al., 2002) (Supplementary Figure 2) clearly suggest that Pab-polD is a thermostable enzyme. These results are interesting since they indicate that denaturation temperature during cycling can be increased when required during PCR. This is particularly useful when genomic DNA contains secondary structures or high GC-rich regions.

As expected, for all three enzymes PCR efficiency was variable in respect to the concentration of the 1.7 million base-pair genomic DNA, with 0.5 ng being the permissive amount for Pab-polB, while Pab-polD and Taq-polA were more sensitive to template dilution. PCR analysis of trace amounts of DNA has become an important concern in forensic investigations (Van Oorschot et al., 2010). While some laboratories set up 0.2 ng as a threshold limit for reliability of the investigations (Budowle et al., 2009), others continue to revise this limit (Kaminiwa et al., 2013). In our conditions, Pab-polD did not behave as an effective tool for the amplification of limited amounts of genomic DNA. Therefore, increasing the number of cycles or varying some compounds within the reaction buffer can be a useful alternative to overcome the limits (Van Oorschot et al., 2010).

In this study, the highest amount of genomic DNA has been applied to all PCR experiments and in these conditions the 0.5 kb target was significantly amplified by Pab-polD, Pab-polB, and Taq-polA. However, upon increasing the length of the target, the differences in PCR performance became more obvious (Pab-polB > Taq-polA > Pab-polD). A maximum of length of 2.95 kb was produced by Taq-polA and Pab-polD. Although barely detectable, Pab-polB could amplify the 4.15 kb target. The difficulty of Pab-polD to PCR amplify long DNA fragments was not due to a high GC content of the DNA regions since all exhibited a value below 48% (Table 1). Pab-polD is known to be endowed with lower processivity than Pab-polB, requiring the PCNA (Proliferating Cell Nuclear Antigen) clamp for robust DNA synthesis (Henneke et al., 2005). Thus, further optimization of PCR amplification of large DNA fragments is certainly possible, for instance, by altering the reaction buffer components, adding PCNA or mixing the two *Pab* PCR enzymes.

Full annealing between primer and template sequences is generally considered crucial for the specific amplification of a nucleic acid sequence (Ghadessy et al., 2004). PCR-based amplification of specific sequences is essential in detecting single nucleotide polymorphisms (SNPs), in identifying microbial-archaeal populations and in diagnostics (Sipos et al., 2007; Liu et al., 2012). In these approaches, “universal” primer sets are used with the possibility to induce the formation of mismatched base pairs at template-primer 3'-termini. As a result, PCR amplification is reduced or fully inhibited (Huang et al., 1992), depending on the length of base mispairs. In our study, Pab-polD was challenged in PCR with mismatched base pairs (1, 2, 3, and 4 base mispairs) at template-primer 3'-termini. Pab-polD could amplify the 0.5 kb DNA target despite the presence of 2 mismatches but with reduced efficiency. Taq-polA retains activity in the presence of 1 mismatch at the 3'-terminus as already published (Huang et al., 1992) but was inhibited by two mismatches. Pab-polB was functional even in the presence of 4 mismatches. The *Pab*PCR enzymes show a higher tolerance to the presence of mismatches which must be attributed to their associated 3'–5' exonuclease function as already compared (Gueguen et al., 2001). Up to four and two mismatches can be accommodated into the exonuclease active site of Pab-polB and Pab-polD, respectively, which subsequently induce the degradation of the 3'-termini. Families B and D from *Pfu* are also known to efficiently process 3'-end termini of primers (Richardson et al., 2013a,b).

Time-dependent PCR extension has been carried out with Pab-polD and compared to Pab-polB and Taq-polA. A PCR product of constant length (1.95 kb) was amplified by increasing the extension time. Under these conditions, Pab-polD was the slowest enzyme able to generate the 1.95 kb target in 6 min whereas 5 and 4 min where required respectively for Taq-polA and Pab-polB. The calculated extension rate of Pab-polD was 0.33 kb/min. This is almost comparable with the value of 0.39 kb/min for Taq-polA and slightly lower to that of 0.48 kb/min for Pab-polB. The values confirmed that Pab-polB, like other family B DNA polymerases, e.g., *Pfu* and *Tfu*, are particularly slow enzymes (Perler et al., 1996; Terpe, 2013). Although the extension rate has been determined by conventional end-point PCR which is not the method of choice compared to real-time quantitative PCR (Arezi et al., 2003), Pab-polD also shows a reduced elongation rate in PCR. In Pab-polB, and potentially Pab-polD, this property could be explained by the slow kinetic partitioning of the primer in the exonuclease and polymerase active sites allowing the DNA polymerase to proofread the nucleotide incorporation events, and when required to remove the misincorporated base (Gouge et al., 2012). On the other hand, the presence of secondary structures could also impede the efficiency of DNA synthesis by the *Pyrococcus* enzymes (Henneke, 2012).

The negative effect of inorganic and organic substances on PCR efficiency and specificity by Pab-polD along with Pab-polB and Taq-polA has been investigated. The DNA polymerase the most resistant to ions NaCl and CaCl_2_ was Pab-polD. This higher resistance to elevated NaCl concentrations is similar to that found for some bacterial and archaeal PCR enzymes investigated previously (Al-Soud and Radstrom, 1998). The highest tolerance to calcium ions compared to other thermostable DNA polymerases (Al-Soud and Radstrom, 1998) places Pab-polD as a suitable enzyme in the amplification of food (e.g., milk and cheese) and human samples (e.g., teeth and bones) for instance. Introduced during the procedure of genomic extraction or naturally present in food and in environmental samples (Charbonnier et al., 1995; Wilson, 1997), phenolic compounds (Ethanol, phenol, and isopropanol) reduced the PCR performance of Pab-polD. These negative effects are commonly observed with most PCR enzymes (Rossen et al., 1992), except for Pab-polB (shown in this study) and *Tth* (*Thermus thermophilus*) (Katcher and Schwartz, 1994). Compounds, such as the SDS anionic detergent and EDTA, known to have direct and indirect negative effects on proteins, respectively, did not dramatically impact on PCR performances by Pab-polD compared to Pab-polB. Surprisingly, the permissive concentration of the two compounds for Taq-polA were slightly different to those found in another study (Yang et al., 2007), indicating that the source of the enzyme, the DNA target to be amplified and the reaction conditions are important parameters impacting on the issue of the investigation. The inhibitory activity of urea in PCR was observed with Pab-polD at a lower concentration threshold compared to Taq-polA or Pab-polB, and the values obtained with Taq-polA confirmed those previously published (Khan et al., 1991). Organic substances like heparin or humic acid were completely inhibitory to PCR reactions by Pab-polD. The strong inhibitory effect of heparin on all three PCR enzymes has been observed with other thermostable DNA polymerases (Yokota et al., 1999). This is not so surprising since heparin is commonly used as a trapping agent of DNA polymerases in both polymerase assays and chromatography. The effects of two heme blood substances, hemoglobin and hematin, did not impact similarly on PCR performances by Pab-polD. While completely resistant to hemoglobin, Pab-polD was sensitive to hematin but to the same level as Taq-polA. Generally, inhibitory effects by heme compounds appear as a drawback in PCR with thresholds dependent on the PCR enzymes used (Akane et al., 1994; Al-Soud and Radstrom, 2001). In this study, Taq-polA was the most sensitive to hemoglobin.

In conclusion, our results demonstrated for the first time that an archaeal family D DNA polymerase is functional in PCR. PCR performances (rate of DNA synthesis, maximal length of amplification, minimal input genomic DNA, resistance to thermal denaturation during cycling, and PCR amplifications with 3'-end mismatched primers) of Pab-polD appears more comparable to Taq-polA than Pab-polB, but with some valuable properties (e.g., high resistance to thermal denaturation during cycling, amplification with primers containing up to 2 mismatches). In addition, due to its superior resistance to inhibitors than Taq-polA (e.g., calcium ions, sodium chloride, hemoglobin, SDS), Pab-polD could replace the enzyme in some applications. Additional investigations (e.g., PCR fidelity, ability to specifically amplify high GC rich content and degraded genomic DNA) are now required to consider Pab-polD as a suitable PCR enzyme that could overcome the handicap encounter by conventional enzymes that are marketed for PCR.

## Conflict of interest statement

The authors declare that the research was conducted in the absence of any commercial or financial relationships that could be construed as a potential conflict of interest.

## References

[B1] Akane A. Matsubara K. Nakamura H. Takahashi S. Kimura K. (1994). Identification of the heme compound copurified with deoxyribonucleic acid (DNA) from bloodstains, a major inhibitor of polymerase chain reaction (PCR) amplification. J. Forensic Sci. 39, 362–372 8195750

[B2] Al-Soud W. A. Radstrom P. (1998). Capacity of nine thermostable DNA polymerases To mediate DNA amplification in the presence of PCR-inhibiting samples. Appl. Environ. Microbiol. 64, 3748–3753 9758794 10.1128/aem.64.10.3748-3753.1998 PMC106538

[B3] Al-Soud W. A. Radstrom P. (2001). Purification and characterization of PCR-inhibitory components in blood cells. J. Clin. Microbiol. 39, 485–493 10.1128/JCM.39.2.485-493.2001 11158094 PMC87763

[B4] Arezi B. Xing W. Sorge J. A. Hogrefe H. H. (2003). Amplification efficiency of thermostable DNA polymerases. Anal. Biochem. 321, 226–235 10.1016/S0003-2697(03)00465-2 14511688

[B5] Bickley J. Short J. K. McDowell D. G. Parkes H. C. (1996). Polymerase chain reaction (PCR) detection of *Listeria monocytogenes* in diluted milk and reversal of PCR inhibition caused by calcium ions. Lett. Appl. Microbiol. 22, 153–158 10.1111/j.1472-765X.1996.tb01131 8936376

[B6] Biles B. D. Connolly B. A. (2004). Low-fidelity *Pyrococcus furiosus* DNA polymerase mutants useful in error-prone PCR. Nucleic Acids Res. 32, e176 10.1093/nar/gnh174 15601989 PMC545472

[B7] Braithwaite D. K. Ito J. (1993). Compilation, alignment, and phylogenetic relationships of DNA polymerases. Nucleic Acids Res. 21, 787–802 10.1093/nar/21.4.787 8451181 PMC309208

[B8] Budowle B. Eisenberg A. J. Van Daal A. (2009). Validity of low copy number typing and applications to forensic science. Croat. Med. J. 50, 207–217 10.3325/cmj.2009.50.207 19480017 PMC2702736

[B9] Cambon-Bonavita M. A. Schmitt P. Zieger M. Flaman J. M. Lesongeur F. Raguenes G. (2000). Cloning, expression, and characterization of DNA polymerase I from the hyperthermophilic archaea *Thermococcus fumicolans*. Extremophiles 4, 215–225 10.1007/PL00010714 10972190

[B10] Cann I. K. Ishino Y. (1999). Archaeal DNA replication: identifying the pieces to solve a puzzle. Genetics 152, 1249–1267 10430556 10.1093/genetics/152.4.1249 PMC1460685

[B11] Cann I. K. Komori K. Toh H. Kanai S. Ishino Y. (1998). A heterodimeric DNA polymerase: evidence that members of Euryarchaeota possess a distinct DNA polymerase. Proc. Natl. Acad. Sci. U.S.A. 95, 14250–14255 10.1073/pnas.95.24.14250 9826686 PMC24359

[B12] Castrec B. Laurent S. Henneke G. Flament D. Raffin J. P. (2010). The glycine-rich motif of *Pyrococcus abyssi* DNA polymerase D is critical for protein stability. J. Mol. Biol. 396, 840–848 10.1016/j.jmb.2010.01.006 20070946

[B13] Castrec B. Rouillon C. Henneke G. Flament D. Querellou J. Raffin J. P. (2009). Binding to PCNA in Euryarchaeal DNA Replication requires two PIP motifs for DNA polymerase D and one PIP motif for DNA polymerase B. J. Mol. Biol. 394, 209–218 10.1016/j.jmb.2009.09.044 19781553

[B14] Charbonnier F. Forterre P. Erauso G. Prieur D. (1995). Purification of plasmids from thermophilic and hyperthermophilic archaea, in Archaea: A Laboratory Manual, eds Place A. R. Robb F. T. (New York, NY: Cold Spring Harbor Laboratory Press), 87–90

[B15] Chien A. Edgar D. B. Trela J. M. (1976). Deoxyribonucleic acid polymerase from the extreme thermophile *Thermus aquaticus*. J. Bacteriol. 127, 1550–1557 8432 10.1128/jb.127.3.1550-1557.1976 PMC232952

[B16] Cline J. Braman J. C. Hogrefe H. H. (1996). PCR fidelity of pfu DNA polymerase and other thermostable DNA polymerases. Nucleic Acids Res. 24, 3546–3551 10.1093/nar/24.18.3546 8836181 PMC146123

[B17] Cubonova L. Richardson T. Burkhart B. W. Kelman Z. Connolly B. A. Reeve J. N. (2013). Archaeal DNA polymerase D but not DNA polymerase B is required for genome replication in *Thermococcus kodakarensis*. J. Bacteriol. 195, 2322–2328 10.1128/JB.02037-12 23504010 PMC3650531

[B18] Delarue M. Poch O. Tordo N. Moras D. Argos P. (1990). An attempt to unify the structure of polymerases. Protein Eng. 3, 461–467 10.1093/protein/3.6.461 2196557

[B19] Dietrich J. Schmitt P. Zieger M. Preve B. Rolland J. L. Chaabihi H. (2002). PCR performance of the highly thermostable proof-reading B-type DNA polymerase from *Pyrococcus abyssi*. FEMS Microbiol. Lett. 217, 89–94 10.1016/S0378-1097(02)01037-6 12445650

[B20] Eckert K. A. Kunkel T. A. (1991). DNA polymerase fidelity and the polymerase chain reaction. PCR Methods Appl. 1, 17–24 1842916 10.1101/gr.1.1.17

[B21] Fogg M. J. Pearl L. H. Connolly B. A. (2002). Structural basis for uracil recognition by archaeal family B DNA polymerases. Nat. Struct. Biol. 9, 922–927 10.1038/nsb867 12415291

[B22] Ghadessy F. J. Ramsay N. Boudsocq F. Loakes D. Brown A. Iwai S. (2004). Generic expansion of the substrate spectrum of a DNA polymerase by directed evolution. Nat. Biotechnol. 22, 755–759 10.1038/nbt974 15156154

[B23] Gouge J. Ralec C. Henneke G. Delarue M. (2012). Molecular recognition of canonical and deaminated bases by *P. abyssi* family B DNA polymerase. J. Mol. Biol. 423, 315–336 10.1016/j.jmb.2012.07.025 22902479

[B24] Gueguen Y. Rolland J. L. Lecompte O. Azam P. Le Romancer G. Flament D. (2001). Characterization of two DNA polymerases from the hyperthermophilic euryarchaeon *Pyrococcus abyssi*. Eur. J. Biochem. 268, 5961–5969 10.1046/j.0014-2956.2001.02550.x 11722585

[B25] Henneke G. (2012). *In vitro* reconstitution of RNA primer removal in Archaea reveals the existence of two pathways. Biochem. J. 447, 271–280 10.1042/BJ20120959 22849643

[B26] Henneke G. Flament D. Hubscher U. Querellou J. Raffin J. P. (2005). The hyperthermophilic euryarchaeota *Pyrococcus abyssi* likely requires the two DNA polymerases D and B for DNA replication. J. Mol. Biol. 350, 53–64 10.1016/j.jmb.2005.04.042 15922358

[B27] Hogrefe H. H. Cline J. Lovejoy A. E. Nielson K. B. (2001). DNA polymerases from hyperthermophiles. Methods Enzymol. 334, 91–116 10.1016/S0076-6879(01)34461-0 11398488

[B28] Huang M. M. Arnheim N. Goodman M. F. (1992). Extension of base mispairs by Taq DNA polymerase: implications for single nucleotide discrimination in PCR. Nucleic Acids Res. 20, 4567–4573 10.1093/nar/20.17.4567 1408758 PMC334186

[B29] Hubscher U. Spadari S. Villani G. Maga G. (2010). DNA Polymerases: Discovery, Characterization and Functions in Cellular DNA Transactions. Singapore: World Scientific Publishing Co. Pte. Ltd.

[B30] Ijzerman M. M. Dahling D. R. Fout G. S. (1997). A method to remove environmental inhibitors prior to the detection of waterborne enteric viruses by reverse transcription-polymerase chain reaction. J. Virol. Methods 63, 145–153 10.1016/S0166-0934(96)02123-4 9015285

[B31] Ishii K. Fukui M. (2001). Optimization of annealing temperature to reduce bias caused by a primer mismatch in multitemplate PCR. Appl. Environ. Microbiol. 67, 3753–3755 10.1128/AEM.67.8.3753-3755.2001 11472961 PMC93085

[B32] Ishino Y. Komori K. Cann I. K. Koga Y. (1998). A novel DNA polymerase family found in Archaea. J. Bacteriol. 180, 2232–2236 9555910 10.1128/jb.180.8.2232-2236.1998 PMC107154

[B33] Joyce C. M. Steitz T. A. (1994). Function and structure relationships in DNA polymerases. Annu. Rev. Biochem. 63, 777–822 10.1146/annurev.bi.63.070194.004021 7526780

[B34] Kaminiwa J. Honda K. Sugano Y. Yano S. Nishi T. Sekine Y. (2013). Vanadium accelerates polymerase chain reaction and expands the applicability of forensic DNA testing. J. Forensic Leg. Med. 20, 326–333 10.1016/j.jflm.2012.09.006 23622484

[B35] Katcher H. L. Schwartz I. (1994). A distinctive property of Tth DNA polymerase: enzymatic amplification in the presence of phenol. Biotechniques 16, 84–92 8136148

[B36] Khan G. Kangro H. O. Coates P. J. Heath R. B. (1991). Inhibitory effects of urine on the polymerase chain reaction for cytomegalovirus DNA. J. Clin. Pathol. 44, 360–365 10.1136/jcp.44.5.360 1646235 PMC496862

[B37] Kong H. Kucera R. B. Jack W. E. (1993). Characterization of a DNA polymerase from the hyperthermophile archaea *Thermococcus litoralis*. Vent DNA polymerase, steady state kinetics, thermal stability, processivity, strand displacement, and exonuclease activities. J. Biol. Chem. 268, 1965–1975 8420970

[B38] Kornberg A. Baker T. A. (1992). DNA Replication. New York, NY: W. H. Freeman and Company

[B39] Langhorst B. W. Jack W. E. Reha-Krantz L. Nichols N. M. (2012). Polbase: a repository of biochemical, genetic and structural information about DNA polymerases. Nucleic Acids Res. 40, D381–387 10.1093/nar/gkr847 21993301 PMC3245023

[B40] Lawyer F. C. Stoffel S. Saiki R. K. Chang S. Y. Landre P. A. Abramson R. D. (1993). High-level expression, purification, and enzymatic characterization of full-length *Thermus aquaticus* DNA polymerase and a truncated form deficient in 5' to 3' exonuclease activity. PCR Methods Appl. 2, 275–287 8324500 10.1101/gr.2.4.275

[B41] Le Y. Chen H. Zagursky R. Wu J. H. Shao W. (2013). Thermostable DNA ligase-mediated PCR production of circular plasmid (PPCP) and its application in directed evolution via *in situ* error-prone PCR. DNA Res. 20, 375–382 10.1093/dnares/dst016 23633530 PMC3738163

[B42] Lipps G. Rother S. Hart C. Krauss G. (2003). A novel type of replicative enzyme harbouring ATPase, primase and DNA polymerase activity. EMBO J. 22, 2516–2525 10.1093/emboj/cdg246 12743045 PMC156004

[B43] Liu J. Huang S. Sun M. Liu S. Liu Y. Wang W. (2012). An improved allele-specific PCR primer design method for SNP marker analysis and its application. Plant Methods 8, 34 10.1186/1746-4811-8-34 22920499 PMC3495711

[B44] Macneill S. A. Baldacci G. Burgers P. M. Hubscher U. (2001). A unified nomenclature for the subunits of eukaryotic DNA polymerase delta. Trends Biochem. Sci. 26, 16–17 10.1016/S0968-0004(00)01709-6 11165510

[B45] Marsic D. Flaman J. M. Ng J. D. (2008). New DNA polymerase from the hyperthermophilic marine archaeon *Thermococcus thioreducens*. Extremophiles 12, 775–788 10.1007/s00792-008-0181-7 18670731

[B46] Matsui I. Urushibata Y. Shen Y. Matsui E. Yokoyama H. (2011). Novel structure of an N-terminal domain that is crucial for the dimeric assembly and DNA-binding of an archaeal DNA polymerase D large subunit from *Pyrococcus horikoshii*. FEBS Lett. 585, 452–458 10.1016/j.febslet.2010.12.040 21192935

[B47] McCullum E. O. Williams B. A. Zhang J. Chaput J. C. (2010). Random mutagenesis by error-prone PCR. Methods Mol. Biol. 634, 103–109 10.1007/978-1-60761-652-8_7 20676978

[B48] McDonald J. P. Hall A. Gasparutto D. Cadet J. Ballantyne J. Woodgate R. (2006). Novel thermostable Y-family polymerases: applications for the PCR amplification of damaged or ancient DNAs. Nucleic Acids Res. 34, 1102–1111 10.1093/nar/gkj512 16488882 PMC1373694

[B49] Moussard H. Henneke G. Moreira D. Jouffe V. Lopez-Garcia P. Jeanthon C. (2006). Thermophilic lifestyle for an uncultured archaeon from hydrothermal vents: evidence from environmental genomics. Appl. Environ. Microbiol. 72, 2268–2271 10.1128/AEM.72.3.2268-2271.2006 16517686 PMC1393191

[B50] Ohmori H. Friedberg E. C. Fuchs R. P. Goodman M. F. Hanaoka F. Hinkle D. (2001). The Y-family of DNA polymerases. Mol. Cell 8, 7–8 10.1016/S1097-2765(01)00278-7 11515498

[B51] Palud A. Villani G. L'Haridon S. Querellou J. Raffin J. P. Henneke G. (2008). Intrinsic properties of the two replicative DNA polymerases of *Pyrococcus abyssi* in replicating abasic sites: possible role in DNA damage tolerance? Mol. Microbiol. 70, 746–761 10.1111/j.1365-2958.2008.06446.x 18826407

[B52] Pavlov A. R. Pavlova N. V. Kozyavkin S. A. Slesarev A. I. (2004). Recent developments in the optimization of thermostable DNA polymerases for efficient applications. Trends Biotechnol. 22, 253–260 10.1016/j.tibtech.2004.02.011 15109812

[B53] Perler F. B. Kumar S. Kong H. (1996). Thermostable DNA polymerases. Adv. Protein Chem. 48, 377–435 10.1016/S0065-3233(08)60367-8 8791630

[B54] Petruska J. Goodman M. F. Boosalis M. S. Sowers L. C. Cheong C. Tinoco I. Jr. (1988). Comparison between DNA melting thermodynamics and DNA polymerase fidelity. Proc. Natl. Acad. Sci. U.S.A. 85, 6252–6256 10.1073/pnas.85.17.6252 3413095 PMC281947

[B55] Richardson T. T. Gilroy L. Ishino Y. Connolly B. A. Henneke G. (2013a). Novel inhibition of archaeal family-D DNA polymerase by uracil. Nucleic Acids Res. 41, 4207–4218 10.1093/nar/gkt083 23408858 PMC3627576

[B56] Richardson T. T. Wu X. Keith B. J. Heslop P. Jones A. C. Connolly B. A. (2013b). Unwinding of primer-templates by archaeal family-B DNA polymerases in response to template-strand uracil. Nucleic Acids Res. 41, 2466–2478 10.1093/nar/gks1364 23303790 PMC3575838

[B57] Rossen L. Norskov P. Holmstrom K. Rasmussen O. F. (1992). Inhibition of PCR by components of food samples, microbial diagnostic assays and DNA-extraction solutions. Int. J. Food Microbiol. 17, 37–45 10.1016/0168-1605(92)90017-W 1476866

[B58] Rothwell P. J. Waksman G. (2005). Structure and mechanism of DNA polymerases. Adv. Protein Chem. 71, 401–440 10.1016/S0065-3233(04)71011-6 16230118

[B59] Rouillon C. Henneke G. Flament D. Querellou J. Raffin J. P. (2007). DNA polymerase switching on homotrimeric PCNA at the replication fork of the euryarchaea *Pyrococcus abyssi*. J. Mol. Biol. 369, 343–355 10.1016/j.jmb.2007.03.054 17442344

[B60] Saiki R. K. Scharf S. Faloona F. Mullis K. B. Horn G. T. Erlich H. A. (1985). Enzymatic amplification of beta-globin genomic sequences and restriction site analysis for diagnosis of sickle cell anemia. Science 230, 1350–1354 10.1126/science.2999980 2999980

[B61] Schrader C. Schielke A. Ellerbroek L. Johne R. (2012). PCR inhibitors - occurrence, properties and removal. J. Appl. Microbiol. 113, 1014–1026 10.1111/j.1365-2672.2012.05384.x 22747964

[B62] Sharkey D. J. Scalice E. R. Christy K. G. Jr. Atwood S. M. Daiss J. L. (1994). Antibodies as thermolabile switches: high temperature triggering for the polymerase chain reaction. Biotechnology (N.Y.) 12, 506–509 10.1038/nbt0594-506 7764710

[B63] Shen Y. Musti K. Hiramoto M. Kikuchi H. Kawarabayashi Y. Matsui I. (2001). Invariant Asp-1122 and Asp-1124 are essential residues for polymerization catalysis of family D DNA polymerase from *Pyrococcus horikoshii*. J. Biol. Chem. 276, 27376–27383 10.1074/jbc.M011762200 11319225

[B64] Sipos R. Szekely A. J. Palatinszky M. Revesz S. Marialigeti K. Nikolausz M. (2007). Effect of primer mismatch, annealing temperature and PCR cycle number on 16S rRNA gene-targetting bacterial community analysis. FEMS Microbiol. Ecol. 60, 341–350 10.1111/j.1574-6941.2007.00283.x 17343679

[B65] Takagi M. Nishioka M. Kakihara H. Kitabayashi M. Inoue H. Kawakami B. (1997). Characterization of DNA polymerase from *Pyrococcus* sp. strain KOD1 and its application to PCR}. Appl. Environ. Microbiol. 63, 4504–4510 9361436 10.1128/aem.63.11.4504-4510.1997 PMC168769

[B66] Terpe K. (2013). Overview of thermostable DNA polymerases for classical PCR applications: from molecular and biochemical fundamentals to commercial systems. Appl. Microbiol. Biotechnol. 97, 10243–10254 10.1007/s00253-013-5290-2 24177730

[B67] Tsai Y. L. Olson B. H. (1992). Rapid method for separation of bacterial DNA from humic substances in sediments for polymerase chain reaction. Appl. Environ. Microbiol. 58, 2292–2295 1386212 10.1128/aem.58.7.2292-2295.1992 PMC195770

[B68] Uemori T. Sato Y. Kato I. Doi H. Ishino Y. (1997). A novel DNA polymerase in the hyperthermophilic archaeon, *Pyrococcus furiosus:* gene cloning, expression, and characterization. Genes Cells 2, 499–512 10.1046/j.1365-2443.1997.1380336.x 9348040

[B69] Van Oorschot R. A. Ballantyne K. N. Mitchell R. J. (2010). Forensic trace DNA: a review. Investig. Genet. 1, 14 10.1186/2041-2223-1-14 21122102 PMC3012025

[B70] Wang Y. Prosen D. E. Mei L. Sullivan J. C. Finney M. Vander Horn P. B. (2004). A novel strategy to engineer DNA polymerases for enhanced processivity and improved performance *in vitro*. Nucleic Acids Res. 32, 1197–1207 10.1093/nar/gkh271 14973201 PMC373405

[B71] Wilson I. G. (1997). Inhibition and facilitation of nucleic acid amplification. Appl. Environ. Microbiol. 63, 3741–3751 9327537 10.1128/aem.63.10.3741-3751.1997 PMC168683

[B72] Wynne S. A. Pinheiro V. B. Holliger P. Leslie A. G. (2013). Structures of an apo and a binary complex of an evolved archeal B family DNA polymerase capable of synthesising highly cy-dye labelled DNA. PLoS ONE 8:e70892 10.1371/journal.pone.0070892 23940661 PMC3733885

[B73] Yamasaki K. Urushibata Y. Yamasaki T. Arisaka F. Matsui I. (2010). Solution structure of the N-terminal domain of the archaeal D-family DNA polymerase small subunit reveals evolutionary relationship to eukaryotic B-family polymerases. FEBS Lett. 584, 3370–3375 10.1016/j.febslet.2010.06.026 20598295

[B74] Yang Y. G. Kim J. Y. Song Y. H. Kim D. S. (2007). A novel buffer system, AnyDirect, can improve polymerase chain reaction from whole blood without DNA isolation. Clin. Chim. Acta 380, 112–117 10.1016/j.cca.2007.01.019 17331487

[B75] Yokota M. Tatsumi N. Nathalang O. Yamada T. Tsuda I. (1999). Effects of heparin on polymerase chain reaction for blood white cells. J. Clin. Lab. Anal. 13, 133–140 10.1002/(SICI)1098-2825(1999)13:3<133::AID-JCLA8>3.0.CO;2-0 10323479 PMC6807949

