# Supplementary material for: PCR performance of a thermostable heterodimeric archaeal DNA polymerase
Source: Front Microbiol. 2014 May 7;5:195. doi: 10.3389/fmicb.2014.00195 (PMC4019886; doi:10.3389/fmicb.2014.00195)

Supplementary Material

PCR performance of a thermostable heterodimeric archaeal DNA polymerase

Tom Killelea [1,2,3], Céline ralec [1,2,3], Audrey Bossé [1,2,3] and Ghislaine Henneke [1,2,3]*

[1] Université de Bretagne Occidentale, UMR 6197, Laboratoire de Microbiologie des Environnements Extrêmes, 29280 Plouzané, France
[2] Ifremer, UMR 6197, Laboratoire de Microbiologie des Environnements Extrêmes, 29280 Plouzané, France
[3] CNRS, UMR 6197, Laboratoire de Microbiologie des Environnements Extrêmes, 29280 Plouzané, France

*** Correspondance:** Ghislaine Henneke, Laboratoire de Microbiologie des Environnements Extrêmes, Ifremer, ZI de la point du diable, CS 10070, 29280 Plouzané, France.
ghenneke@ifremer.fr

1. Supplementary Materials and Methods

Inhibitory effects of organic and inorganic substances on PCR

The PCR amplification of the 0.5 kb DNA target was carried out in the presence of increasing concentrations of each compound as described in the Materials and Methods section of the full manuscript.

Functional thermostability

Thermostability of Pab-polD and Isis has been evaluated in a previous study by measuring DNA polymerase activity after various pre-incubation times at different temperatures as described (Gueguen et al., 2001). The two-hour incubation time values for Pab-polD and *Isis* have been used with permission along with the full data set as shown in Supplementary Figure 2.

2. Supplementary Figures

Supplementary Figure 1. Evaluation of the inhibitory effects of organic and inorganic substances on PCR. The efficiency and sensitivity of PCR amplification by Pab-polD are compared with *Isis* and *Taq*. One representative gel is shown for each condition. The permissive values are indicated in Table 3.

Supplementary Figure 2. Thermostability of *Isis* (A) and Pab-polD (B) at different temperatures.

3. References

Gueguen, Y., Rolland, J.L., Lecompte, O., Azam, P., Le Romancer, G., Flament, D., Raffin, J.P., and Dietrich, J. (2001). Characterization of two DNA polymerases from the hyperthermophilic euryarchaeon *Pyrococcus abyssi*. *Eur J Biochem* 268, 5961-5969. doi: 10.1046/j.0014-2956.2001.02550.x.

Supplementary Figure 1. Evaluation of the inhibitory effects of organic and inorganic substances on PCR

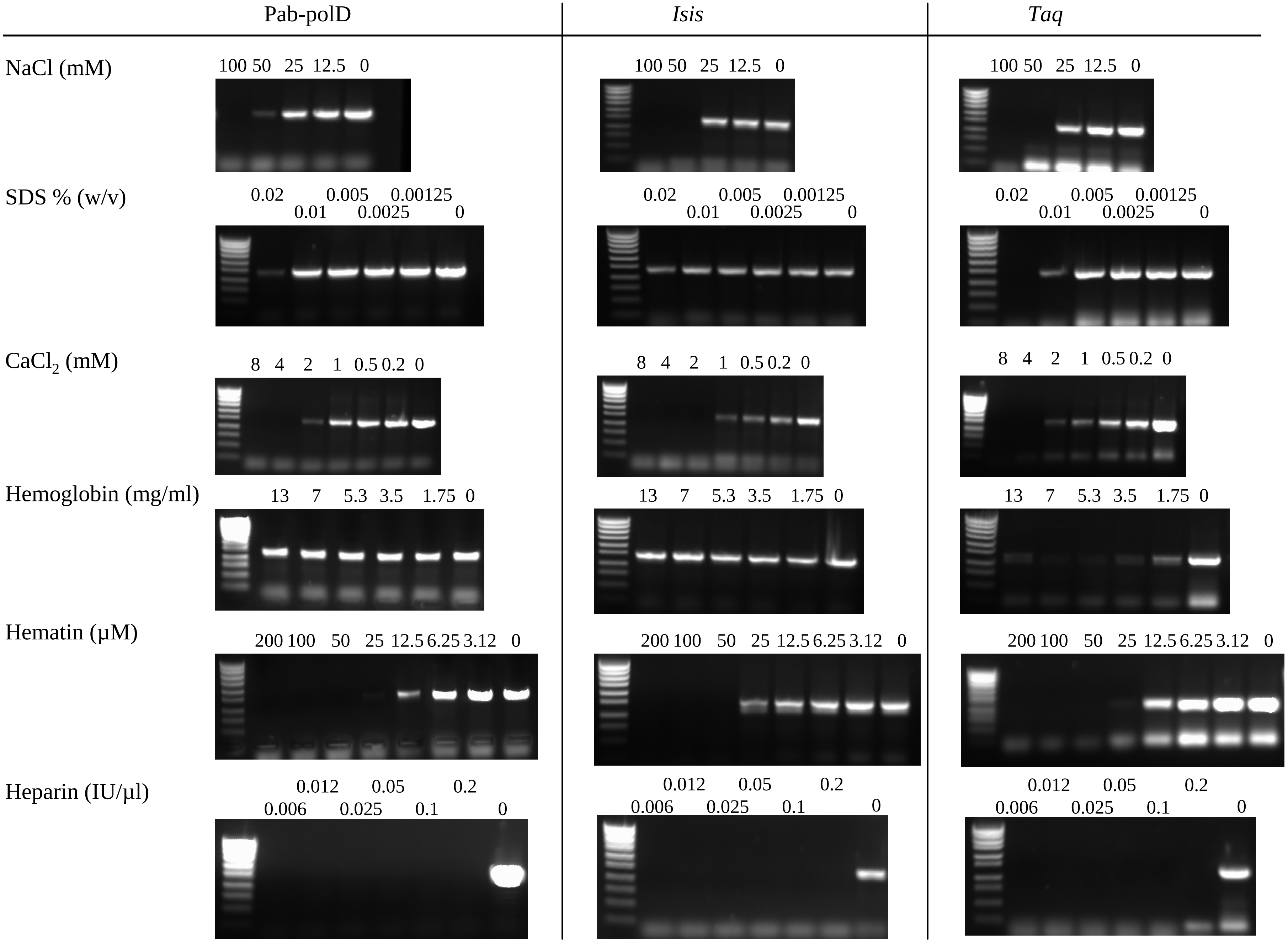

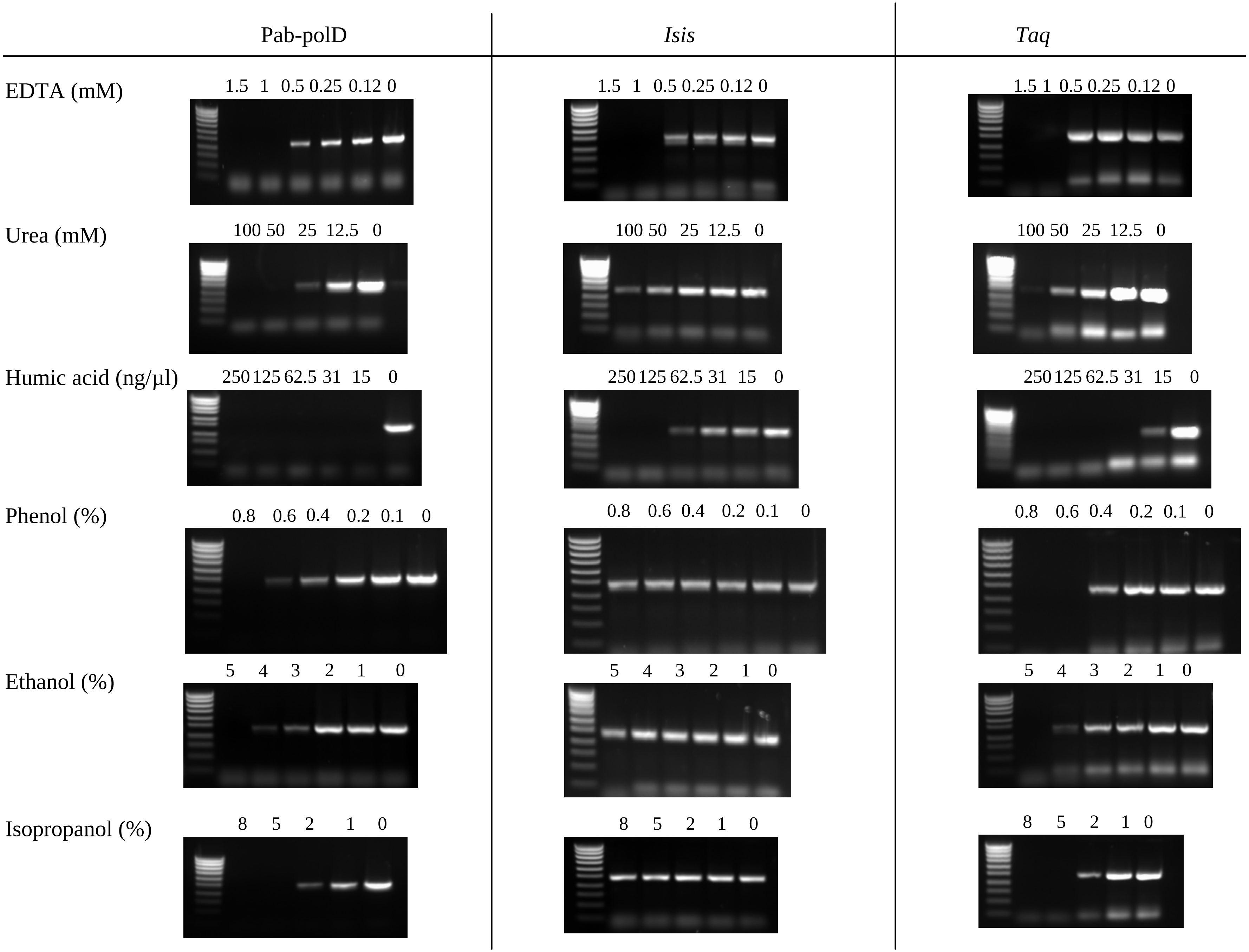

Pab-polD
Isis
Taq
EDTA (mM)
1.5 1 0.5 0.25 0.12 0
Urea (mM)
100 50 25 12.5 0
Humic acid (ng/µl)
250 125 62.5 31 15 0
Phenol (%)
0.8 0.6 0.4 0.2 0.1 0
Ethanol (%)
5 4 3 2 1 0
Isopropanol (%)
8 5 2 1 0

Supplementary Figure 2. Thermostability of Pab-polD and *Isis*

A

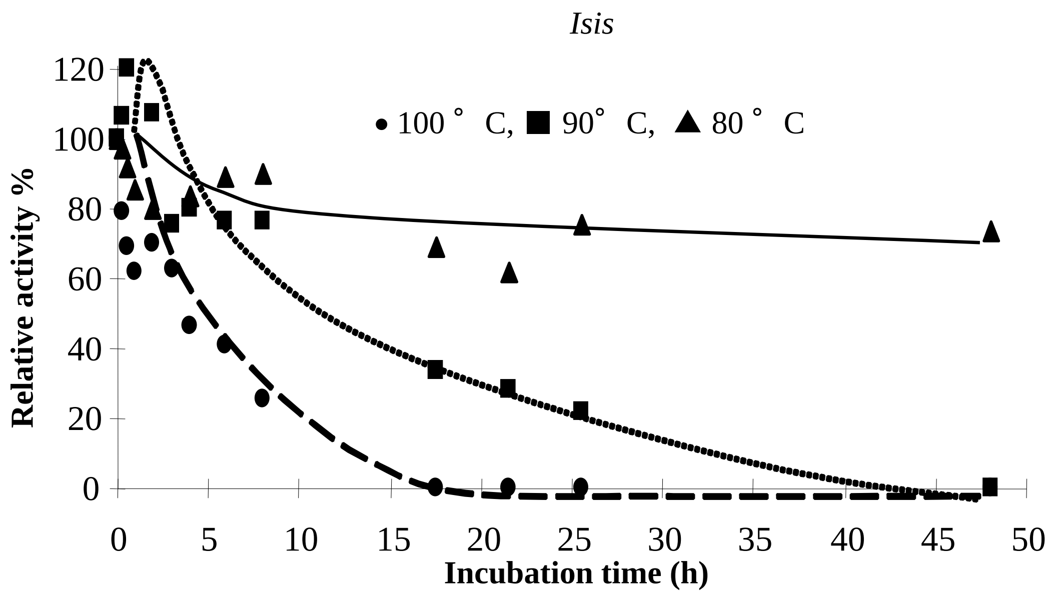

B

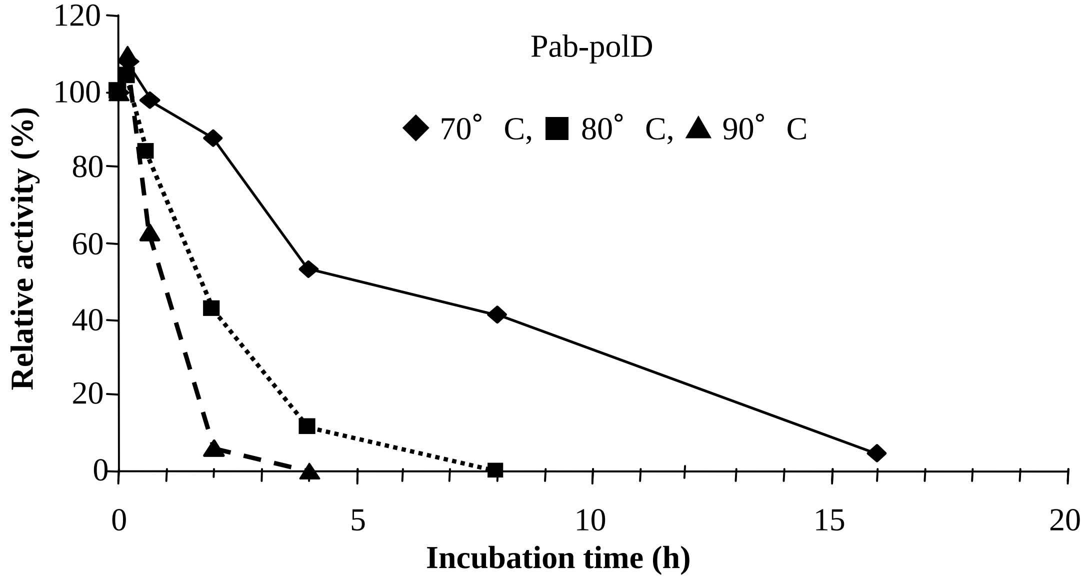

Supplement: Supplementary file 1 [file DataSheet1.PDF]
